# Dynamic Synchronous Capture Algorithm for an Electromagnetic Flowmeter

**DOI:** 10.3390/s17040821

**Published:** 2017-04-10

**Authors:** Yong-Yi Fanjiang, Shih-Wei Lu

**Affiliations:** Department of Computer Science and Information Engineering, Fu Jen Catholic University, New Taipei 24205, Taiwan; sway0929@gmail.com

**Keywords:** electromagnetic flowmeter, Gaussian blur, moving average algorithm

## Abstract

This paper proposes a dynamic synchronous capture (DSC) algorithm to calculate the flow rate for an electromagnetic flowmeter. The characteristics of the DSC algorithm can accurately calculate the flow rate signal and efficiently convert an analog signal to upgrade the execution performance of a microcontroller unit (MCU). Furthermore, it can reduce interference from abnormal noise. It is extremely steady and independent of fluctuations in the flow measurement. Moreover, it can calculate the current flow rate signal immediately (m/s). The DSC algorithm can be applied to the current general MCU firmware platform without using DSP (Digital Signal Processing) or a high-speed and high-end MCU platform, and signal amplification by hardware reduces the demand for ADC accuracy, which reduces the cost.

## 1. Introduction

The electromagnetic flowmeter has been developed over 70 years since the beginning of the 20th century, and it is used in oil, chemical, metallurgy, textile, food, hydraulic building, industrial measurement, and medical industries [1]. Most of the current flowmeter developments are limited to mechanical measurement in Taiwan. There has been no research performed on the use of electromagnetic flowmeters in the industrial measurement field. Their unit price is high, and due to import problems, a significant amount of time is required to obtain them. Frost & Sullivan and the International Energy Agency (IEA) show that the investment in global basic energy equipment will accumulate to 26 quadrillion US dollars over the period from 2007 to 2030, and the intelligent flow meter market is expected to reach 7.76 Billion US dollars by 2022, at a CAGR (Compound annual growth rate) of 5.4% between 2016 and 2022 [2].

In several places in the world, the infrastructures of electric, oil, and natural gas will need to be replaced by 2030. Facing a competitive environment and the demands of global energy conservation and carbon reduction, various industrial users are devoting more attention to the efficiency of production plants to reduce energy consumption as much as possible and thereby improve competitiveness. Therefore, numerous investments, which are used to raise the level of factory automation, gather field data and monitor real time, can enhance the efficiency of the process control system. In oil, gas, and energy industries, custody transfer facilities need reliable flow measurement equipment. In chemical and pharmaceutical industries, electromagnetic flowmeters require high accuracy. Sensors and field devices, including electromagnetic flowmeters, can be developed in diverse ways or manners.

At present, most methods in the electromagnetic flowmeter paper do not specify which algorithm to use in the calculation of the flow. There are many ways to calculate the flow through hardware or sensor architecture improvements, but often it is often found that they use most of the firmware computing platform (MCU) specifications (especially in large quantities using FPGAs [3,4,5,6] or DSP [7] and other high-cost hardware components). Therefore, it can be speculated that other methods must be obtained through the more powerful hardware specifications to calculate, resulting in cost increases and also a lack of flexibility. In view of this, it is very practical research to study how to improve the efficiency of algorithm implementation and related signal processing to reduce hardware dependency.

This paper proposes a dynamic synchronous capture (DSC) algorithm to calculate the flow rate for an electromagnetic flowmeter. This implementation platform is based on a 32-bit microcontroller unit (MCU), which is inexpensive and easily obtained. This algorithm can meet the requirements for real-time calculations and for conversions of a correct and accurate flow rate signal. Moreover, it can simultaneously avoid noise interference [8]. When calculating the flow rate signal, this algorithm can combine imaging algorithms and statistical methods, which are used to increase the S/N ratio (signal-to-noise ratio) in the limited resources firmware platform to obtain effective results.

## 2. Background

### 2.1. Faraday’s Law

The principle of the electromagnetic flowmeter is based on Faraday’s law of induction, which states that if a conductor is moved through a magnetic field, then a voltage that is proportional to the velocity of the conductor will be induced. In accordance with the law of induction, a voltage *U* can be induced in the process liquid that is proportional to the flow velocity $\bar{v}$ of the process liquid, induction *B* and the inside tube diameter *D* as follows:

(1) $$U=\frac{d\phi }{dt}=\frac{BdA}{dt}=B\frac{Ddl}{dt}=BD\bar{v}$$

where *B* is the magnetic flux density (Wb/m^2^), $\bar{v}$ is the average speed of the conductive liquid motion (m/s), *D* is the diameter of the pipe (m), *dl* is the conductive liquid diameter (m), *A* is the cross-sectional area (m^2^), and *U* is the signal electromotive force (V).

When Equation (1) is applied to the electromagnetic flowmeter, a calibration factor *K* is applied, and the signal electromotive force *U* can be expressed as follows [9]:

(2) $$U=KBD\bar{v}$$

where *K* is a non-dimensional constant.

The signal voltage *U* is picked up by electrodes that have conductive contact with the process liquid and insulation from the pipe wall [10]. Using

(3) $$Q_{V}=\left(\frac{\pi }{4}D^{2}\right)\cdot \bar{v}$$

the signal voltage *U* can be converted by a signal converter into a flow indication *Q_V_* as follows:

(4) $$Q_{V}=\frac{\pi }{4}\cdot D\cdot \frac{U}{KB}$$

Furthermore, the flow rate can be converted into standardized signals appropriate to the process. Here, $Q_{V}$ is the volume flow rate (m^3^/s).

This process indicates that the nominal pipe diameter *D* is a constant value. When the magnetic flux density changes, the flow rate is directly proportional to the signal electromotive force *U*. Thus, we can observe that the flow measurement of the electromagnetic flowmeter is independent of other physical parameters, which is one of its advantages. Moreover, the above formula was established, and the limited conditions are satisfied from the following section.

### 2.2. Assumptions and Limitations

The electromagnetic flowmeter needs to satisfy the following conditions:

1. Assumption 1: Axis-symmetrical velocity distribution. The profile of the flow velocity is assumed to have an axis-symmetrical distribution. The induced current in the liquid and the electric field are symmetrical and parallel to the axial direction of the liquid.
2. Assumption 2: Uniform, constant distributed magnetic field. The magnetic field is assumed to have a constant and uniform distribution. If this assumption is true, then it can be ignored by the effects of the conductive liquid in the magnetic field generated by movement, i.e., the effect of the induced current on the magnetic field distribution and the effect of the interaction between the induced current and the electromagnetic force with the liquid flow velocity. Both of these effects in the measurement of the liquid metal are not negligible.
3. Assumption 3: Non-magnetic liquid. The measured liquid is assumed to be a non-magnetic liquid, and its permeability *μ* is consistent with the permeability of the vacuum *μ*_0_. Thus, the effect of the interaction between the magnetic liquid and the magnetic field work for the flow measurement can be ignored. It can be observed that the measured fluid flow holds under the above conditions and hypothesis, and the induced electromotive force *U* can be considered proportional to the instantaneous volume flow rate *Q_V_*. Their relationship is completely linear.
4. Assumption 4: Uniform and isotropic liquid conductivity. The conductivity of the liquid is assumed to be uniform and isotropic. It is independent of the electric field or the liquid flow.

### 2.3. Excitation Mode

The different excitation methods used for measuring different liquids in various environments have diverse effects and functions.

#### 2.3.1. DC Excitation

The coils of the electromagnetic flowmeter can be powered by either alternating current (AC) or direct current (DC) [11]. The DC excitation uses the direct-current voltage to provide the electromagnetic flowmeter with a steady voltage so that the excitation of the magnetic field can be steady. The DC excitation is reliable and simple. Additionally, it is rarely affected by the electric supply. The problem with the direct-current excitation is that it leads to electric polarization, which results in a weaker flow signal [12]. The electrode’s resistance becomes more significant, and the electric supply drifts at the same time [13,14].

#### 2.3.2. Sinusoidal Wave Excitation

The sinusoid excitation skills can directly replace the direct-current excitation. It can eliminate the surface electric polarization, as well as decrease the influence of the electric charge drift and the internal resistance of the electromagnetic flowmeter [15]. The orthogonal interference of the amplitude and frequency are in direct proportion. Furthermore, they cause same-phase interference and lead to the electromagnetic flowmeter, which does not have drift disadvantages.

#### 2.3.3. Pulsed DC Excitation Low-Frequency Rectangular Wave Excitation

The electromagnetic flowmeter is used for low frequency rectangle excitation, whose frequency is that of the electric supply frequency reduced from 1/4 to 1/10 [14]. The DC excitation does not cause eddy currents, orthogonal interferences, same-phase interferences and non-electric polarizations. The signal’s amplification is used for the calculation. This steady excitation can avoid zero drift. Additionally, it can tolerate noise well. It is disadvantageous that the amplitude of the differential interference is directly proportional to the frequency.

## 3. Requirements of DSC Algorithm Design

**Figure 1:**
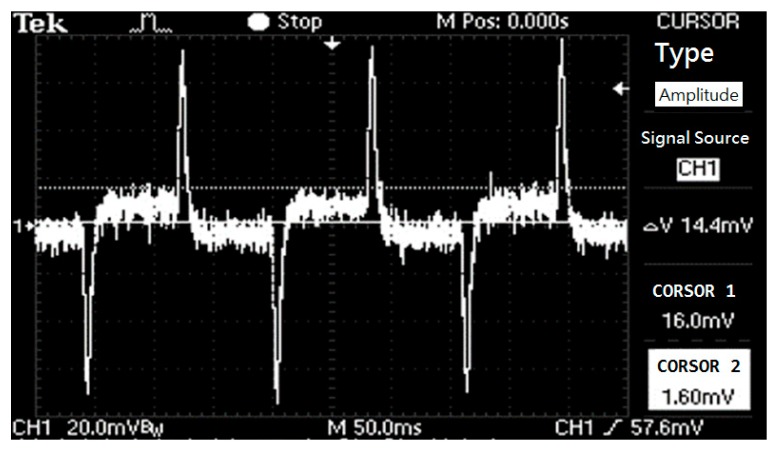
Flow signal waveforms.

The coil generates the excitation signal and is sensed by the signal at both ends of the electrode, that is, the so-called flow rate original signal Figure 1, by calculating the size of the vibration to calculate the instantaneous flow rate, the flow rate and the signal amplitude. Because the power level sensed out of the signal is very small (mV), it is very susceptible to interference and will produce differential interference phenomena that need to be overcome. The following steps illustrate how to calculate and challenge the project.

### 3.1. Calculating the Flow Rate Signal

As illustrated in Figure 1, the flow signal’s change depends on the amplitude wave of the excitation at the moment it senses. The flow signal is proportional to the amplitude of the excitation wave. We should avoid the differential interference when calculating the amplitude [16]. The differential interference occurs as a result of the excitation direction of the coil, which is varying. Furthermore, the differential interference has a fixed size based on the excitation current. The flow signal is larger, and the excitation signal’s amplitude is larger as well. Additionally, it can cover the wave of differential interference.

### 3.2. Comparison of Different Flow Rate Signals

For the high and low excitation waves of the flow signal, it is apparent that the excitation waves are different. The flow signal is high due to the large excitation signal’s amplitude. The differential interference will be covered by the signal. In a low flow velocity, the excitation will produce differential interference signals, so we need to simultaneously conform to the high and low flow signals’ algorithm.

### 3.3. Noise Suppression

The electromagnetic flowmeter’s signal arises from sensing the voltage, which requires accurate quality. Noise can lead to mistakes in the electromagnetic flowmeter, and any mistakes will affect the repetition.

### 3.4. Level Flutter and Offset

The high and low excitation waves of the flow signal and their zero-voltage level located in the signal are different. From the data, if positive and negative values are used to calculate the positive and negative half cycles, it will cause unusual results and detect mistakes. The zero-voltage level has different reactions for different flows. When we observe the steady flow signals, the signals will follow the zero-voltage level up and down, which indicates that the flow signal can move up and down. Based on the analysis above, in this paper, a DSC algorithm has been designed to provide the following functionalities:

- An algorithm for calculation of the flow rate signal based on the characteristics of the electromagnetic flowmeter;
- Avoidance of noise suppression and the differential interference’s sector while calculating the amplitude;
- Signal interpretation, as well as dynamic and real-time computation;
- Greater accuracy and repeatability for the current solution; and
- Consistency for productized and repetitive flow production.

## 4. Implementation

### 4.1. Hardware Architecture

The flow signal’s algorithm needs to have the flow signal features mentioned in the previous section. Furthermore, the algorithm needs to be able to calculate the flow signal and address the noise, signal drift, and unsteady voltage level. Moreover, the algorithm can be implemented in the common 32-bit MCU. We can calculate the flow signal effectively, but we also need to use the filter, which can cause problems related to noise, the signal’s movement, and the level. The algorithm needs to be implemented in the current. Thus, its efficiency has to conform to the MCU’s consumptive resources. The algorithm also requires additional work to function (e.g., communication, display, and key functions) and to produce the real-time signal detection and calculation.

**Figure 2:**
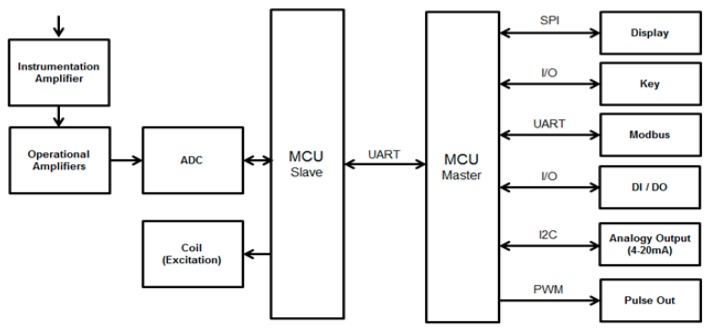
Hardware block diagram.

Figure 2 illustrates the hardware block of the MCUs in the DSC. The MCU master is responsible for controlling the UI (user interface). The LCM (Liquid Crystal Module) signifies that the detection of the key adds the analogy output. The flow signal enters from the sensor. Because its signal is extremely small, the highest flow amplitude voltage depends on the mV. The instrumentation amplifier creates a larger signal, which becomes even larger when passing the OPA (Operational Amplifier). The signal passes the ADC (analog to digital converter), and it is selected by the MCU slave afterwards. It produces the flow signal’s detection and the algorithm’s calculation.

### 4.2. Excitation Circuit

When designing the excitation, the suitable excitation will be selected and analyzed. This paper selects a low frequency rectangle excitation 0 and saddle-shaped excitation coils [17].

**Figure 3:**
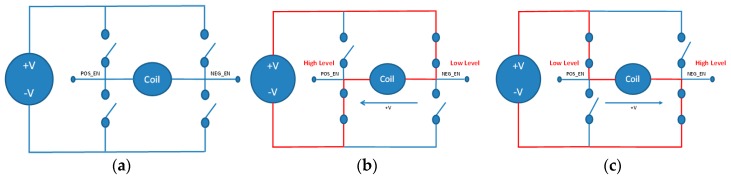
(**a**) H-Bridge circuit; (**b**) High voltage POS_EN and low voltage NEG_EN; (**c**) Low voltage POS_EN and high voltage NEG_EN.

The H-Bridge circuit allows the DC electric motor to have forward, reverse and stop functions. Figure 3a illustrates the H-Bridge circuit. Figure 3b indicates that when the POS_EN has a high voltage and the NEG_EN has a low voltage, due to the cross conduction of the transistor, the path of the excitation current moves to the left. However, Figure 3c indicates that when the POS_EN has a low voltage and the NEG_EN has a high voltage, the path of the excitation current moves to the right.

### 4.3. DSC Algorithm

When designing the excitation, the suitable excitation will be selected and analyzed. This paper selects a low frequency rectangle excitation of 0 and saddle-shaped excitation coils [17].

#### 4.3.1. Coil Excitation Sync

Using the firmware I/O POS, the NEG pin transmits the H-Bridge to produce the magnetic field’s change and to lead to the differential interference’s signal. The differential interference’s signal is used to ensure that the synchronous flow signal starts and calculates the flow signal accurately. Obtaining the synchronous signal can consume the MCU resources and lead to burdens because certain signals need to operate and calculate. To avoid these problems, we can use the H-Bridge circuit.

#### 4.3.2. Start Sampling Data

Based on the Nyquist sampling theorem, the sampling rate (*f_s_*) must be twice as large as that of the highest frequency test signal. For example, the algorithm takes a 1 kHz signal and brings the duty to 160 ms (6.25 Hz). The signal is converted and stored in the memory of the MCU when the synchronization signals appear. The stored data are the original flow rate signals. Based on the synchronization signals, the data in the MCU can be categorized into positive and negative cycles. Therefore, they can be processed in the next stage of the flow rate’s calculation algorithm.

We need to consider the problem related to the data’s switch. When the signal is switching, it requires the following steps: the signal is detected, obtained, and switched. It is easy to add the system’s burden. The switching frequency is 1 kHz, which corresponds to a period of 1 ms. The process of loading the ADC and switching the effective figure through the MCU cannot be completed in 1 ms. Therefore, we use the queue to obtain and switch the signals.

**Figure 4:**
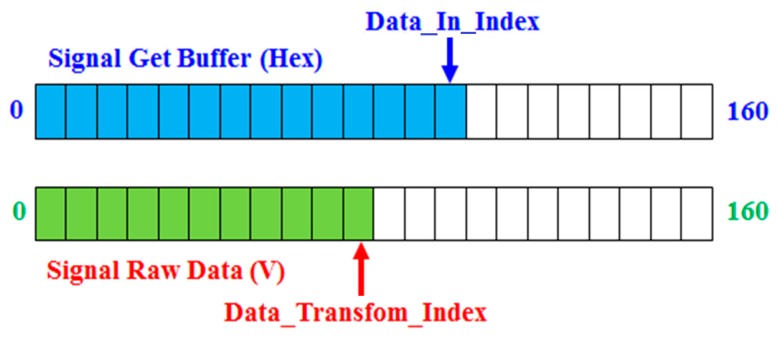
Using queue conversion data.

As illustrated in Figure 4, when conducting the original flow rate signal acquisition, the counter Data_In_Index will increase after obtaining a flow rate signal. This process is repeated until the entire signal cycle is fetched (i.e., 160 slots). When the Data_In_Index increases, the Data_Transform_Index is used to check whether the signal needs to be converted. The advantage of this approach is that it allows the signal to be acquired and converted into parallel processing, and thus, it saves time and improves the conversion efficiency. Additionally, it can avoid the inability to detect the flow rate in real time due to the delay of signal acquisition.

#### 4.3.3. Obtain Available and Conversion Data

**Figure 5:**
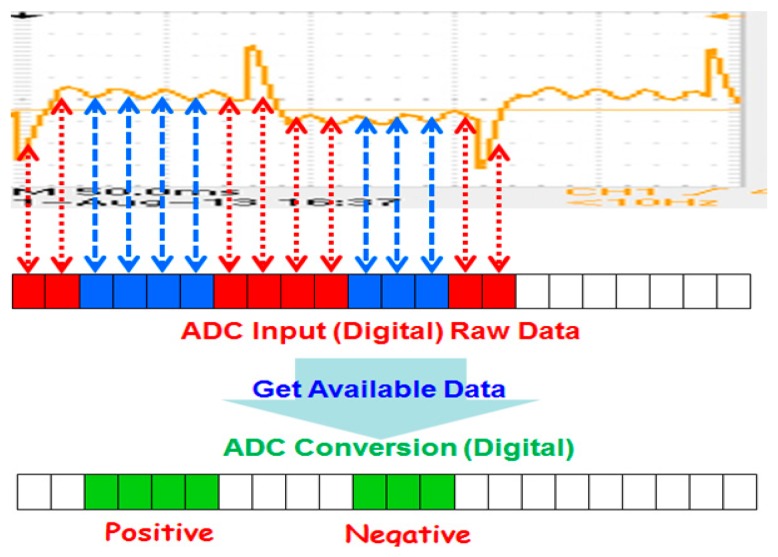
Sampling flow signal.

After the synchronous signal, the distribution of time can be obtained. When each signal has been sampled, it can judge whether the signal is located in the effect zone. When the signal is in the effect zone, it will process the signal’s switch. This approach can avoid significantly unsteady zones or the non-effect zone. For example, the differential interference’s pulses decrease several noise and error signals. They reduce the MCU burden when attempting to detect signals simultaneously. The positive half-cycle and the negative half-cycle (see Figure 5) can also be recognized.

#### 4.3.4. Average to Positive/Negative Region

The signals of the positive half-cycle and the negative half-cycle will be averaged, which can advance and calculate the flow signal’s amplitude. Because the positive half-cycle and the negative half-cycle do not signify positive and negative, the flow amplitude has to use the absolute value of the data. The result of the calculation is raw data.

Using Equation (5), we can calculate how many data are in the positive zone and in the negative zone. We attempt to calculate the data separately and equally.

(5) $$\Delta F=\left|1/p\sum_{k=0}^{p-1}P\left[k+p\right]-1/n\sum_{k=0}^{n-1}N\left[k+n\right]\right|$$

where *p* is the number of total positive signal points, n is the number of total negative signal points, *P*[ ] is the positive input signal, and *N*[ ] is the negative input signal.

#### 4.3.5. MAQ (Moving Average + Quick Sort) of Flow Results

When switching the original flow signal, the data are first saved in the array. When the new data enter, the old data are deleted as the new data are saved. The average is not calculated in this step because the signal will most likely add noise or error signals. If the average of the signals affects the S/N ratio directly, their accuracy will decrease.

**Figure 6:**
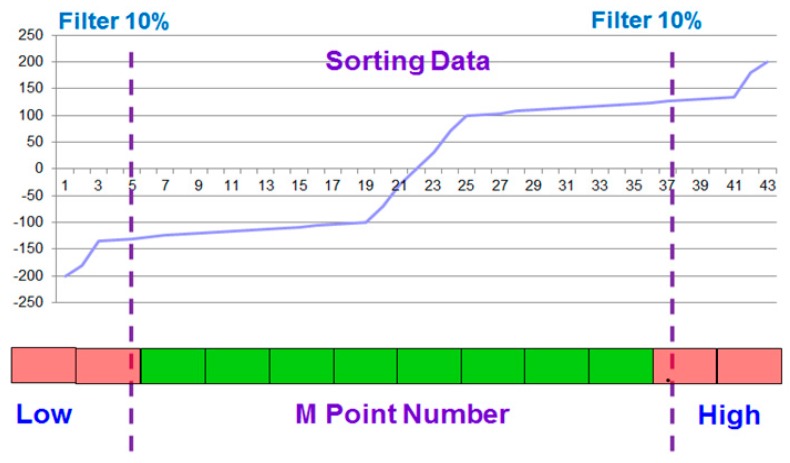
Algorithm of MAQ (Quicksort and Average).

After arranging the flow signal in the order of its values, it can eliminate the environment that causes potential noise. The signal’s data persist from 10% to 90%. The lower bound (10%) is the differential interference time. After filtering, the remaining data are used to calculate the totals and averages, which can be observed in Figure 6. By adding steady signals and calculating the averages, we can obtain the flow signals.

## 5. Experiments and Results

### 5.1. Flow Testing Environment

**Figure 7:**
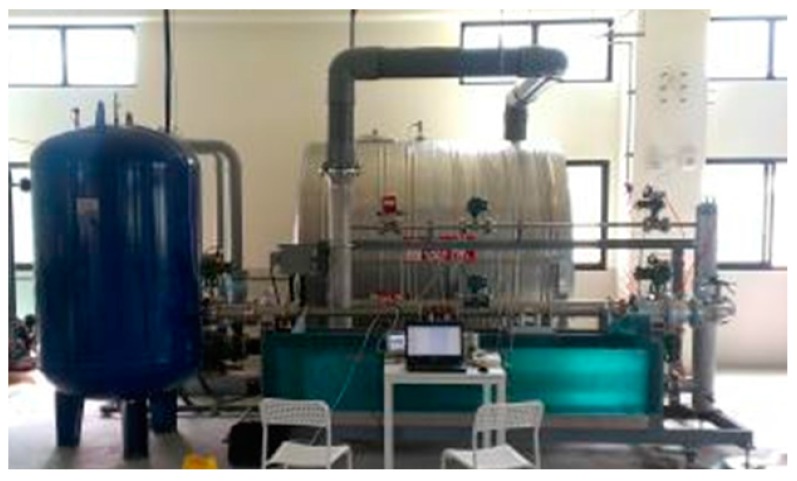
Flow testing apparatus.

Figure 7 depicts the flow testing apparatus in this study. The testing equipment uses the pump to generate the flow. When the pressure tanks accumulate pressure and water level, a valve regulates the flow of water and installs the electromagnetic flowmeter to generate the flow test condition, flow rate monitor, and control. Thus, the tests must obey the national flow measurement rules. The upstream pipe length is less than 5D (D is the nominal pipe diameter), and the downstream pipe length is greater than 2D.

Because of limitations caused by various factors and the interference test environment, the test standard uses a comparison method after obtaining the test results, which is a comparison to a flow meter (referred to as the standard) at the national flow calibration laboratory with standard parts and standard conditions. The pulse output is used as the cumulative amount of traffic statistics.

The specifications of the flow test environment are listed below:

- the diameter of the testing flowmeter is DN80 (nominal diameter 80 mm);
- the dynamic range of the flow rate is 0 to 10 m/s;
- the testing liquid is water;
- the standard flowmeter uses a Yokogawa AXF (0.2%).

These experiments indicate that the results of the proposed DSC algorithm, ΔF (raw data of flow rate), Gaussian (Gaussian filter algorithm—using mean filter of third order), and MA (moving average algorithm) can calculate the average of the flow, and then the process can be repeated [18]. The experimental results for ΔF+MA, ΔF+MA+Gaussian, and the proposed DSC are listed in Table 1, Table 2 and Table 3, respectively.

### 5.2. Algorithm Comparisons

**Figure 8:**
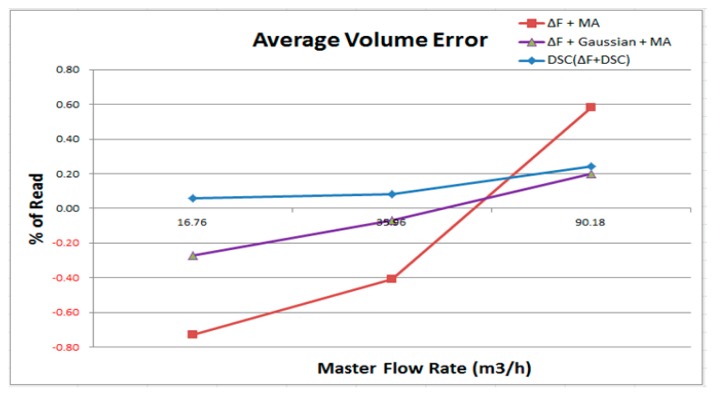
Comparison with ΔF+MA, ΔF + MA + Gaussian, and DSC for average volume error.

Figure 8 shows the average volume error of the flow rate from low (16.76 m^3^/h) to high (90.18 m^3^/h); the lower error rate indicates that the performance of the meter is better and more accurate. In the results, ΔF + MA is the most unstable output because, at different flow rates, it has a high deviation and non-linearity.

**Figure 9:**
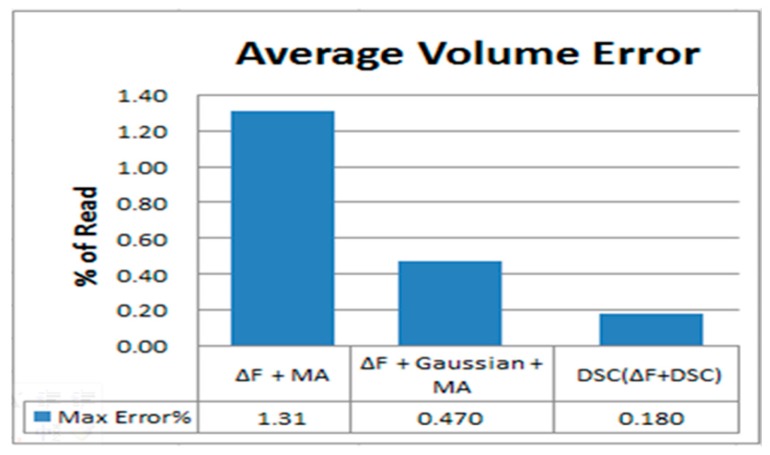
Maximum error rate of ΔF + MA, ΔF + MA + Gaussian, and DSC for average volume error.

The results indicate that with a Gaussian filter, the accuracy is significantly better than that of ΔF + MA (see ΔF + MA + Gaussian). Lastly, the results indicate that the DCS has greater accuracy than the above two calculation methods. As illustrated in Figure 9, the DSC method has the best effect. Its max error is 0.18%. The second-best method uses ΔF + MA + Gaussian, whose max error is 0.47%. Lastly, ΔF + MA has a max error of 1.31%.

**Figure 10:**
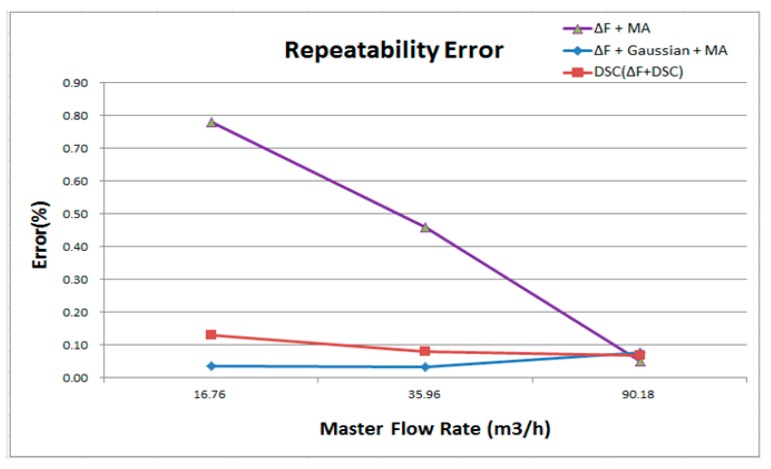
Comparison with ΔF + MA, ΔF + MA + Gaussian, and DSC for repeatability error.

**Figure 11:**
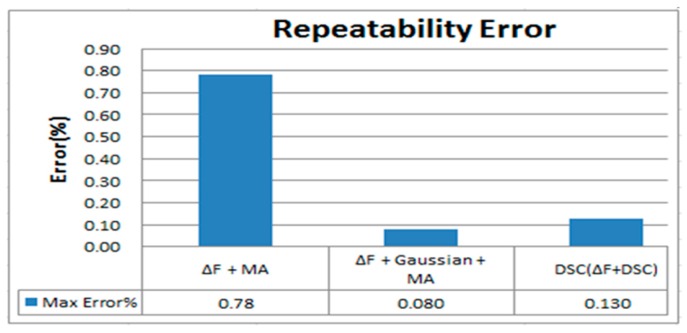
Maximum error rate of ΔF + MA, ΔF + MA + Gaussian, and DSC for repeatability error.

The repetitive error rate represents the stability of the flow meter measurement. A lower error rate indicates that the flow meter uses a more stable detection for each measurement in the same testing environment. Based on Figure 10 and Figure 11, the maximum error of using the ΔF + MA + Gaussian is approximately 0.08%.

Using of ΔF + MA + Gaussian has a better result can be expected because the Gaussian algorithm blurs images to achieve a smooth effect. In other words, the Gaussian uses a large number of average operations to achieve better repeatability error results, however, it also means that even if the signal has interferences or noises, the Gaussian is still to smoothing the calculation, so that the real flow signal will be affected by the smoothing calculation, thereby reducing the accuracy. This is why the Gaussian filter is out of consideration in this study. Figure 11 shows that the maximum error of using DSC is 0.13%, which is extremely close to that of ΔF + MA + Gaussian (0.08%). Lastly, the ΔF + MA has a maximum error of 0.78%.

The above test data and statistical results indicate that our proposed DSC algorithm has better performance than the other two algorithms in both the average volume error and the repeatability error. Additionally, the results indicate that using the Gaussian filter benefits the repeatability. However, the algorithm can still be used even though it has interference and noise. The true flow signals and the accuracy will be affected; therefore, this research does not consider the Gaussian filter.

### 5.3. Compared with Krohne-IFC100

The experimental results are compared with the Krohne IFC 100, a known measurement device from Germany. The device’s accuracy is listed at 0.5% in the specifications. The same transducer is used for calculating the average volume error and repeatability.

**Figure 12:**
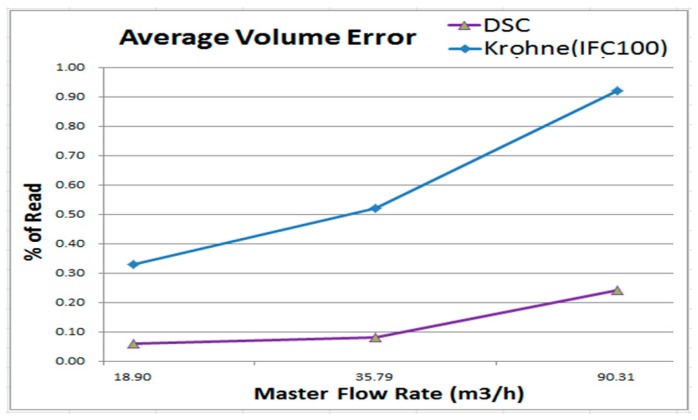
Comparison with Krohne IFC 100 for average volume error.

**Figure 13:**
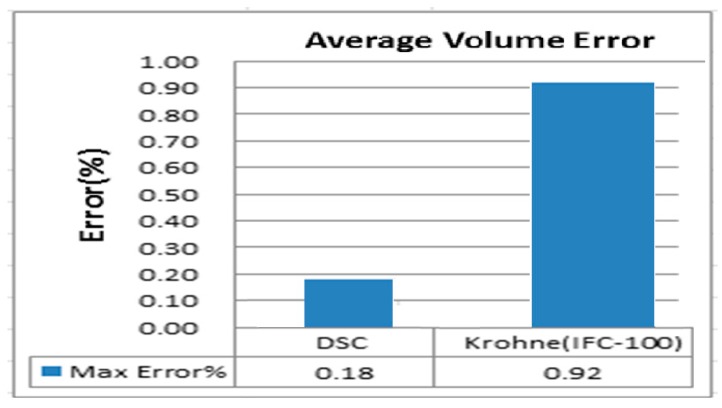
Maximum error rate of DSC and Krohne IFC 100 for average volume error.

Figure 12 depicts the average volume error comparison between the DSC and Krohne (IFC 100, 0.5%). The results indicate that, for the Krohne device (IFC 100, 0.5%), the error in the higher flow rates is higher; the rate is better in the low flow. However, the error in the DSC is more stable and lower on average. The average volume error trend of the DSC is poor in the areas with high flow rates and better in the areas with low flow rates, which is similar to the Krohne device. As illustrated in Figure 13, the max average volume error is 0.18 percent. The Krohne device’s (IFC 100, 0.5%) value is 0.92%.

**Figure 14:**
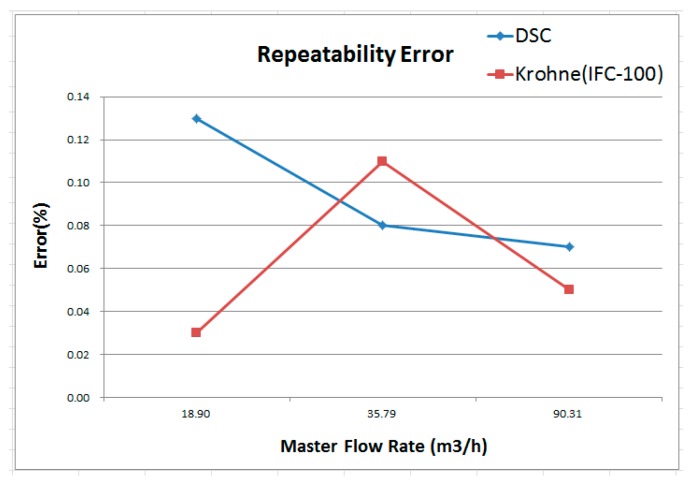
Comparison with Krohne IFC 100 for repeatability error.

**Figure 15:**
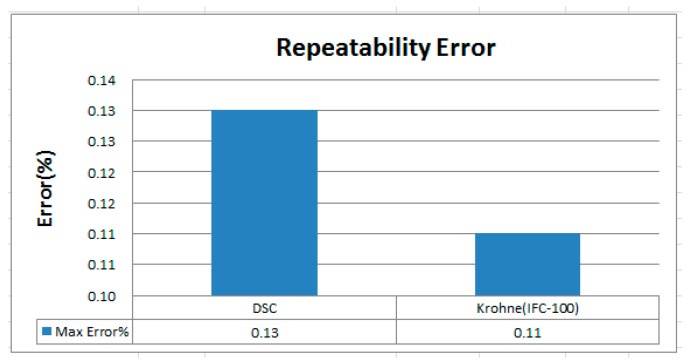
Maximum error rate of DSC and Krohne IFC 100 for repeatability error.

Figure 14 depicts comparison result of repeatability error between DSC and Krohne (IFC-100, 0.5%). It can be found that the repeatability error of Krohne (IFC-100, 0.5%) is higher in the middle flow rate, while the regional high flow rate repeatability is better. However, the repeatability error of DSC in this study is more stable to the higher flow. It can be found in the high and low flow rate area, the performance of Krohne (IFC-100, 0.5%) is more excellent, while the flow rate section is better for this study. Figure 15 shows the maximum reproducibility data, which can be found to be about 0.13% in this study and 0.11% for Krohne (IFC-100, 0.5%). Although Krohne (IFC-100, 0.5%) showed slightly better reproducibility of only about 0.02%, repeatability is very close.

### 5.4. Comparison with Wavelet Transform

The experimental results illustrate a comparison of the wavelet transform algorithm [19]. The wavelet transform uses the H-Bridge and the excitation. The processor used is LPC2136 (ARM7TDMI-S based high-performance 32-bit RISC Microcontroller, Philips, Tokyo, Japan). Although the test flow’s range is not completely the same, we obtain a condition to determine an average flow rate statistic that is accurate. Using the wavelet transform to perform the switching maneuver, the flow is approximately 0.45 to 2.9 m/s. After the switch becomes linear, the flow speed has a 0.3% inaccuracy.

**Figure 16:**
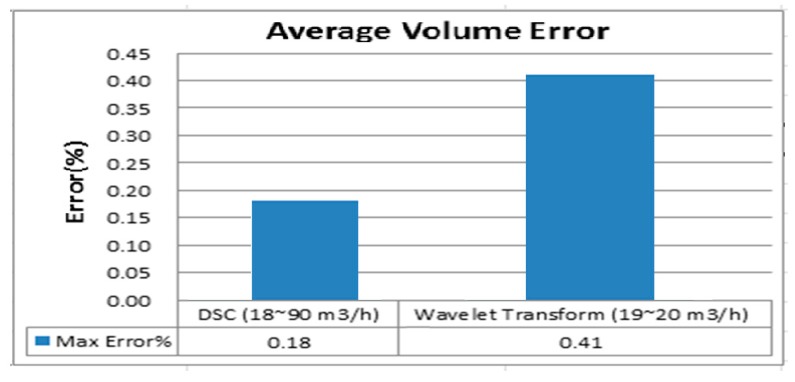
Comparison with wavelet transform for average volume error.

As illustrated in Figure 16, we use the wavelet transform algorithm to develop a comparison of the average flow rate. In order to ensure the objectivity and consistency of the data, we try to compare the same flow rate as much as possible. Using the test data of the wavelet transform algorithm, the test flow rate is limited between 19–20 m^3^/h, and the maximum average flow error is 0.41% (0.26% + 0.16%).

Compared with the DSC, its measurement range is approximately a flow speed of 1 m/s to 5 m/s. This measurement range can cover the wavelet transform algorithm. Furthermore, its accuracy is better than that of other algorithms.

### 5.5. Processor Price Comparison

**Figure 17:**
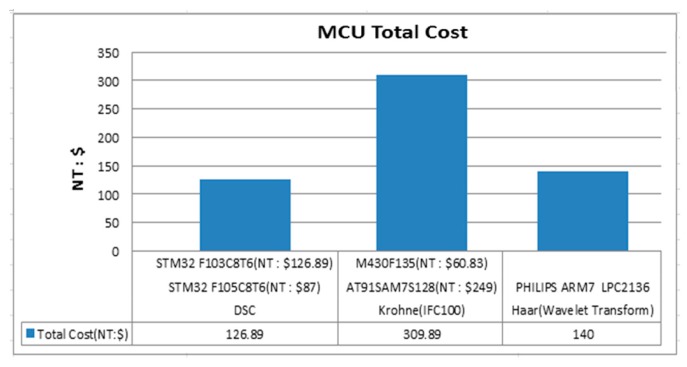
Cost comparison.

As shown by the prices provided in Figure 17 of the processor cores for the MCU, the price of the DSC is nearly 2.5 less than that of the Krohne (IFC 100, 0.5%) and approximately 10% less than that of the wavelet transform algorithm; however, the average volume flow rate and the reproducible performance of the DSC are not inferior to those of the Krohne (IFC 100, 0.5%) and the wavelet transform algorithms. Table 4 summarizes the results of the proposed DSC algorithm in comparison with the other algorithms. From Table 4, it can be noted that the proposed DSC algorithm has the smallest average volume error and total cost. The repeatability error of the proposed DSC algorithm is comparable to that of the Krohne IFC 100 but is smaller than that of the other algorithms.

## 6. Conclusions

In this paper, a dynamic synchronous capture algorithm is proposed to calculate the flow rate for an electromagnetic flowmeter. From the experiment, the DSC algorithm fits the flow speed detection’s algorithm, and the DSC algorithm can be implemented in the mainstream 32-bit MCU. The results indicate that the data are less than those of the Krohne device (IFC 100, 0.5%). Its measurement range can cover the wavelet transform switch algorithm. Furthermore, its accuracy is greater than that of the wavelet transform switch algorithm, and the DSC algorithm can obtain better competition in the market. The algorithm does not need a DSP or MCU; thus, it is suitable for the application platform and occasion.

The only condition of the DSC algorithm is its ability to be used with a simple H-Bridge circuit for control. In addition to reducing costs, the algorithm’s effectiveness to meet the specifications of the meter can be compared to the cost to provide ADC signal resolution, which will allow it to obtain higher accuracies and better competition in the market. In this paper, which used only water as a measurement standard, suggestions for future research are to continue studies using different liquids of various amounts in detection tests. Changes to the signal are likely to occur, especially with the additional use of different excitation frequencies, and other digital filters can also be assessed to calculate the flow rate signal.

## Figures and Tables

**Table 1:** Experimental result of the ΔF + MA.

Upstream Temp (°C)	Downstream Temp (°C)	Master Flow Rate (m^3^/h)	Master Total Volume (L)	Test Total Volu58me (L)	Volume Error (% of Read)	Repeatability Error (%)	Average Volume Error (% of Read)
31.36	31.66	90.18	1491.40	1499.79	0.56	0.05	0.58
31.28	31.58	90.22	1491.70	1499.68	0.53		
31.29	31.58	90.07	1484.10	1493.70	0.65		
31.20	31.41	35.96	594.80	595.97	0.20	0.46	−0.41
31.20	31.41	35.93	595.10	589.55	−0.93		
31.20	31.41	36.00	598.60	595.66	−0.49		
31.12	31.33	16.76	278.20	274.21	−1.43	0.78	−0.73
31.12	31.33	16.80	278.00	278.99	0.36		
31.12	31.33	16.74	278.40	275.28	−1.12		

**Table 2:** Experimental results of the ΔF+MA+GAUSSIAN.

Upstream Temp (°C)	Downstream Temp (°C)	Master Meter Flow Rate (m^3^/h)	Master Meter Volume (L)	Test Meter Volume (L)	Volume Error (% of Read)	Repeatability Error (%)	Average Volume Error (% of Read)
38.96	39.41	17.69	591.90	590.40	−0.25	0.036	−0.27
38.97	39.41	17.81	591.40	590.00	−0.24		
38.89	39.33	17.77	589.70	587.90	−0.31		
38.80	39.17	35.80	1190.00	1189.50	−0.04	0.035	−0.07
38.72	39.16	35.84	1191.50	1190.80	−0.06		
38.64	39.08	35.75	1188.20	1186.90	−0.11		
38.56	39.00	88.42	2941.30	2947.50	0.21	0.077	0.20
38.55	38.99	88.55	2942.50	2950.70	0.28		
38.48	38.91	88.55	2954.70	2958.40	0.13		

**Table 3:** Experimental results of the DSC.

Upstream Temp (°C)	Downstream Temp (°C)	Master Flow Rate (m^3^/h)	Master Total Volume (L)	Test Total Volume (L)	Volume Error (% of Read)	Repeatability Error (%)	Average Volume Error (% of Read)
29.21	29.37	88.95	1469.15	1473.66	0.31	0.07	0.24
29.21	29.37	88.89	1471.11	1475.04	0.27		
29.21	29.29	89.09	1463.51	1465.70	0.15		
29.13	29.29	37.42	619.59	619.47	−0.02	0.08	0.08
29.13	29.29	37.42	619.05	620.13	0.17		
29.13	29.30	37.52	619.21	619.80	0.10		
29.05	29.29	19.00	314.45	314.93	0.15	0.13	0.06
29.05	29.30	19.01	314.33	313.93	−0.13		
29.04	29.21	19.00	314.40	314.91	0.16		

**Table 4:** Summary of results between DSC algorithm in comparison with the other algorithms.

	ΔF+MA	ΔF + MA + Gaussian	Krohne (IFC-100)	Wavelet Transformation [19]	DSC
Average Volume Error (%)	1.31	0.47	0.92	0.41	0.18
Repeatability Error (%)	0.78	0.08	0.11	n/a	0.13
Total Cost (NT dollar)	n/a	n/a	390.89	140	126.9
